# Penetrating Keratoplasty in a Resource-Constrained Country: Surgical Outcomes and Challenges in the Absence of a Domestic Eye Bank

**DOI:** 10.7759/cureus.95711

**Published:** 2025-10-29

**Authors:** Nuzhat Rahil, Mahnoor A Khan, Muhammad A Malik, Bilal Khan, Ahmad Hanan, Rahil Malik

**Affiliations:** 1 Ophthalmology, Medical Teaching Institution, Lady Reading Hospital, Peshawar, PAK; 2 Ophthalmic Surgery, Khyber Medical College, Peshawar, PAK; 3 Ophthalmology, Medical Teaching Institution, Khyber Medical College/Khyber Teaching Hospital, Peshawar, PAK; 4 Vitreal Surgery, Khyber Eye Foundation, Peshawar, PAK

**Keywords:** bullous keratopathy, corneal dystrophy, donor cornea, keratoconus, penetrating keratoplasty

## Abstract

Background: Corneal blindness is a significant public health issue worldwide, particularly in resource-constrained countries with limited access to specialized ophthalmic care. Keratoplasty is one of the essential treatment approaches for rehabilitating vision in patients with corneal pathologies.

Aims: The primary aim of the study was to assess the challenges faced during penetrating keratoplasty (PK) and the outcomes of surgeries in a resource-constrained country in the absence of a domestic eye bank. The secondary aim was to assess the outcomes of PK.

Methods: This cross-sectional descriptive study included patients undergoing PK for various corneal pathologies at the Department of Ophthalmology, Medical Teaching Institute, Lady Reading Hospital (MTI-LRH), Peshawar, Pakistan, between January and March 2025.

Results: PK was performed in 42 patients (27 (64.3%) male and 15 (35.7%) female), with a mean age of 33.9 ± 20.9 years. The right eye was involved in 16 (38.1%) patients, while bilateral cases were 13 (31.0%). The leading cause for PK was keratoconus with very low best corrected visual acuity (BCVA) (n = 15, 35.7%), followed by non-traumatic corneal opacity. The waiting period for surgery was >20 months in 31 (73.8%) patients. After PK, the patients who achieved BCVA >6/60 increased from two (4.8%) at one-day follow-up to 29 (69.1%) at six-month follow-up. The major challenge in PK was a lack of surgical tools in 33 (78.6%) patients and a delay in donor tissue availability in 30 (71.4%) patients.

Conclusion: Visual acuity was significantly improved after PK, particularly at long follow-up periods. The major challenge in PK was the lack of surgical tools and the delay in donor tissue availability.

## Introduction

Corneal blindness is a leading public health problem worldwide, particularly in resource-poor countries with limited access to specialized ophthalmology care [1]. The burden of corneal blindness is high in Pakistan, affecting around 250,000 individuals [2]. This is mainly caused by infectious keratitis, trauma, bullous keratopathy, corneal dystrophies, and keratoconus [3].

Keratoplasty is an essential treatment approach to rehabilitate vision in patients having corneal opacity and related pathologies [4,5]. Keratoplasty is an umbrella term covering a set of surgical interventions, including penetrating keratoplasty (PK), deep anterior lamellar keratoplasty (DALK), and Descemet’s membrane endothelial keratoplasty (DMEK). Yet, the success of keratoplasty in underresourced places like Pakistan is hampered by limited donor corneas, poor surgical facilities, insufficiently trained staff, and financial constraints affecting both patients and the healthcare system [6]. Among these, the inadequacy of donor corneas presents a major obstacle to corneal transplantation [7]. This deficiency is compounded by the absence of a robust eye banking system and low public awareness regarding cornea donation [8]. Cultural and religious beliefs sometimes impede the donation of corneal tissue, resulting in dependence on imported corneas; however, this may prove impractical due to financial and logistical challenges [9].

Despite these issues, there is evidence that keratoplasty is feasible in Pakistan. For example, a retrospective study demonstrated a 30-month graft survival among 90% keratoconus patients [10]. Moreover, it was concluded that encouraging outcomes can be attained with careful patient selection (i.e., patients meeting the defined inclusion criteria) and surgical experience [11].

Identification of specific challenges encountered when performing keratoplasty in a low-resource hospital of a low and middle-income country (LMIC) is a clinically relevant and important arena of ophthalmology. The aim of this study was to assess the challenges faced during PK in a resource-constrained country in the absence of a domestic corneal bank and relying on donor tissue from other countries. By identifying the primary obstacles and determining which practices work best, it may be possible to find ways to improve access to and the success of keratoplasty in underprivileged regions. It may be noted that this work has not been previously presented anywhere.

## Materials and methods

This prospective cross-sectional study was performed at the Department of Ophthalmology, Medical Teaching Institute, Lady Reading Hospital (MTI-LRH), Peshawar, Pakistan. The study was conducted between January and March 2025, with follow-up visits at one day, one month, and six months. The study-hosting hospital is a tertiary care center that handles referrals from the entire province of Khyber Pakhtunkhwa and the neighboring country of Afghanistan. To perform PK, MTI-LRH relies solely on the donation of corneal tissue from the Association of Physicians of Pakistan Descent of North America (APNNA), a United States (US)-based organization. In general, there is limited availability of corneal tissue from APNNA, and consequently, patients may wait for months before undergoing surgical intervention.

All patients advised for PK due to corneal pathologies were included in the study, with no restrictions on gender, age, ethnicity, or geographical location. However, patients advised for lamellar keratoplasty and those with incomplete medical records were excluded.

Patients were registered after a complete ocular examination, which included slit lamp examination, intraocular pressure measurement, posterior segment examination, and B-scan (ocular ultrasound), where indicated, as well as assessments for any systemic comorbidities. Additionally, fitness for general anesthesia was evaluated, as it is required for the planned/tentative surgery. After evaluating the patients for enrollment, the donor organization (APNNA) was contacted for corneal donation. Once the cornea was received, surgery was performed under sterile conditions by two surgeons. Briefly, after anesthesia administration, the diseased corneal tissue was excised using a trephine (Surgistar, Vista, US). The donor corneal graft was then precisely positioned and sutured, with attention to wound apposition and suture tension to ensure optimal graft adhesion and minimize postoperative astigmatism. All patients were prescribed postoperative topical antibiotics and topical steroids. Follow-up was conducted on days 1, 30, and 180 postoperatively, hereafter referred to as follow-ups 1, 2, and 3, respectively.

The variables for collected data included demographics (age and gender), indication for PK, method, quality, and date of expiry of the donor cornea. Preoperative and postoperative best corrected visual acuity (BCVA), preoperative and postoperative challenges, including the waiting period for the graft, preoperative and postoperative complications, and instances of lost to follow-up were recorded.

IBM SPSS Statistics version 25 (IBM Corp., Armonk, US) was used for statistical analysis. In particular, categorical variables such as gender, diagnosis, and challenges faced were assessed for frequency and percentage, while scale variables like age and visual acuity were presented as mean and standard deviation (SD). Statistical significance was determined using the Student's t-test for normally distributed variables and the Mann-Whitney U test for other variables.

Ethical approval for this study was obtained from the Institute Ethical Review Board. All patients provided written consent for participation in this study.

## Results

PK was performed on a total of 42 patients. Among these, 27 (64.3%) were male, while 15 (35.7%) were female. The mean age of the patients was 33.90 ± 20.92 years. Details of the age distribution are shown in Table 1.

**Table 1:** Age-distribution of the patients included in this study

Demographic variable		Number of patients (percentage)
Gender	Male	27 (64.3)
	Female	15 (35.7)
Age (years)	≤10	03 (7.2)
	11-20	13 (31)
	21-30	07 (16.7)
	31-40	04 (9.5)
	41-50	03 (7.2)
	51-60	08 (19.1)
	>60	04 (9.5)

**Figure 1:**
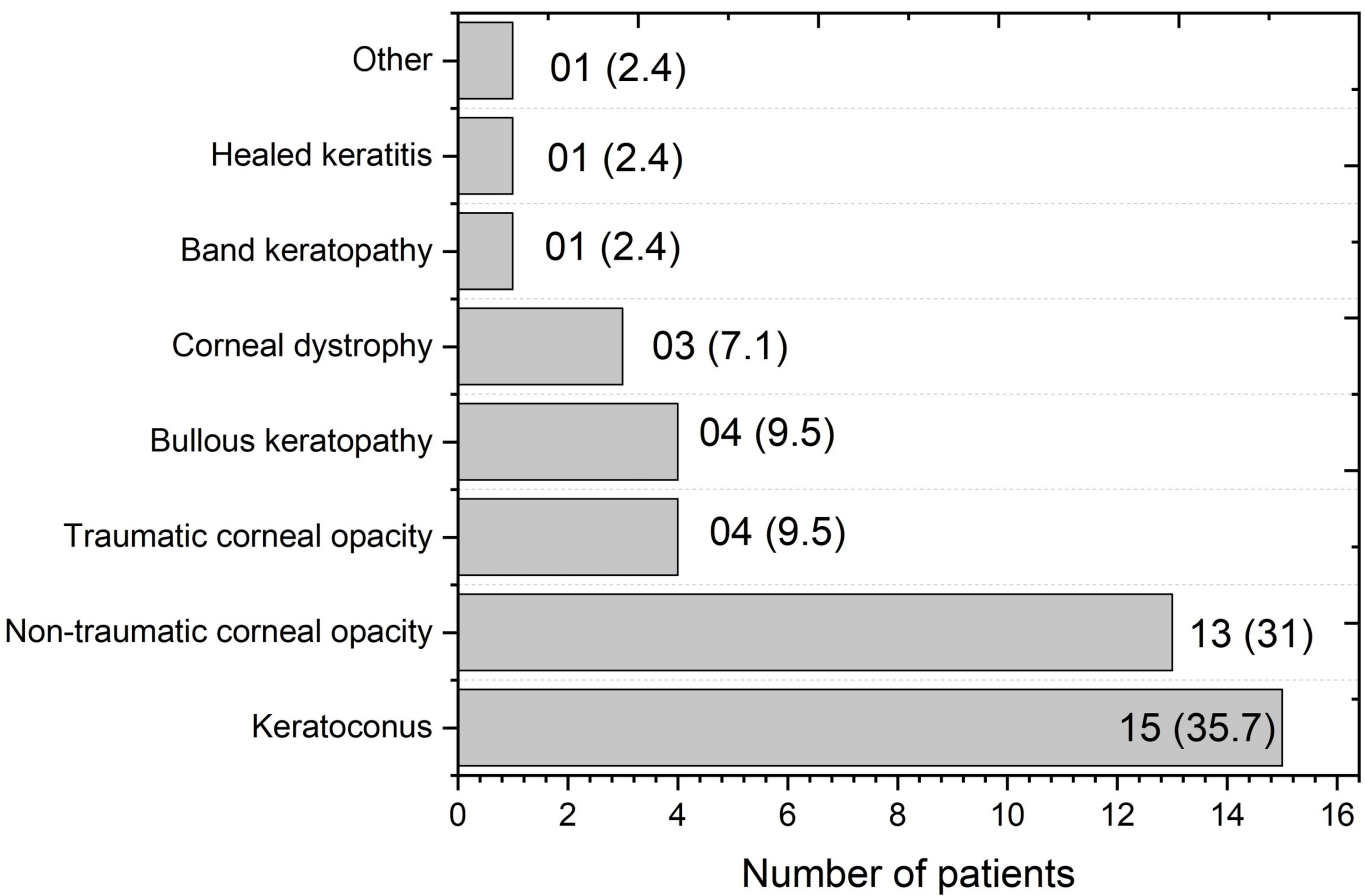
Preoperative corneal pathologies Numbers in parentheses indicate percentages.

Involvement of the right eye by the clinical pathology team was observed in 16 (38.1%) patients, while 13 (31.0%) patients presented with bilateral pathologies. When analyzing the different pathologies of cornea and indications of PK, the leading cause was keratoconus, affecting 15 (35.7%) patients, as illustrated in Figure 1.

The donated corneas were received in a preservation medium (i.e., Eusol C medium) at a controlled temperature of 2-8 °C to maintain tissue viability. During transportation, a specialized cold-chain courier system was used to prevent temperature fluctuations. Upon delivery to the transplant center, these corneas were transplanted within 24 hours of receipt.

The waiting period before surgery was more than 20 months in 31 (73.8%) patients, while seven (16.7%) patients waited for 15-20 months for the arrival of corneas. The BCVA on the Snellen chart before surgery was 6/60 in 12 (28.6%) patients, 11 (26.2%) patients had visual acuity of counting fingers close to the eye, and 10 (23.8%) had a visual acuity of 1/60 (see Table 2). BCVA progressively improved over six months, as evident in consecutive follow-ups. The proportion of patients who achieved BCVA >6/60 increased from two (4.8%) at the first follow-up to 29 (69.1%) on the third follow-up, as summarized in Table 2. It is important to note that since 10 (23.8%) patients were lost to follow-up, these statistics are based on the remaining 32 patients. Furthermore, Table 3 describes the visual acuity data as assessed using LogMAR.

**Table 2:** Comparison of BCVA on Snellen chart at the baseline and follow-ups 1, 2, and 3 PL: perception of light; HM: hand movement; CF: counting fingers; BCVA: best corrected visual acuity

Visual acuity	Number of patients (percentage)			
	Baseline	Follow-up 1	Follow-up 2	Follow-up 3
PL	03 (7.1)	03 (7.1)	01 (2.4)	01 (2.4)
HM	04 (9.5)	15 (35.7)	03 (7.1)	02 (4.8)
CF (close to eye)	11 (26.2)	12 (28.6)	02 (4.8)	0
1/60	10 (23.8)	10 (23.8)	03 (7.1)	0
6/60	12 (28.6)	02 (4.8)	13 (31)	05 (11.9)
6/36	02 (4.8)	-	19 (45.2)	09 (21.4)
6/24	-	-	01 (2.4)	14 (33.4)
6/18	-	-	-	01 (2.4)
6/12 or better	-	-	-	0
Lost to follow-up	-	-	-	10 (23.8)

**Table 3:** Comparison of LogMAR BCVA at the baseline and follow-ups 1, 2, and 3 *p-value calculated with the Mann-Whitney U test and refers to the comparison of measurements at each follow-up with the baseline. BCVA: best corrected visual acuity

Assessment	Visual acuity	p-value*	Mean differences
Baseline	1.71 ± 0.64	-	-
Follow-up 1	1.60 ± 0.42	0.2	0.11 ± 0.55
Follow-up 2	1.18 ± 0.51	<0.001	0.53 ± 0.57
Follow-up 3	0.84 ± 0.46	<0.0001	0.87 ± 0.60

**Figure 2:**
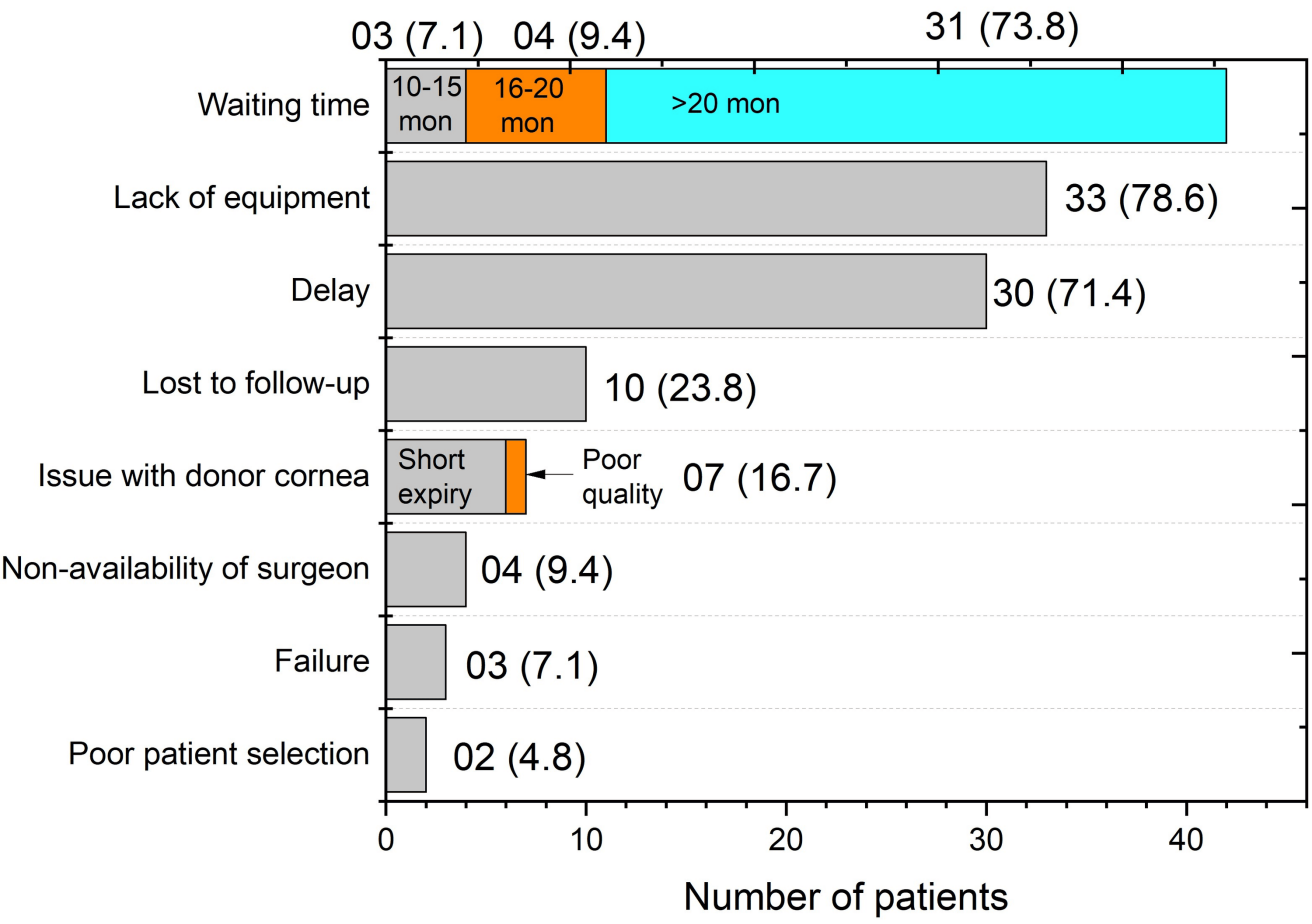
Summary of challenges faced by the patients Numbers in parentheses indicate percentages. mon: months

The challenges faced during PK were multifactorial, as shown in Figure 2. The most common challenge was the lack of surgical tools, such as trephines, affecting 33 (78.6%) patients. Additionally, the delay in donor tissue availability due to the unavailability of a local corneal bank was a major factor in 30 (71.4%) patients. The proportion of patients lost to follow-up was also notable, at 10 (23.8%).

None of the patients presented with graft rejection during follow-ups; however, graft failure (i.e., loss of transparency) was observed in three (7.1%) patients. No patients experienced infection or endophthalmitis.

When analyzing the postoperative complications as part of the outcomes, 10 (23.8%) patients were lost to follow-up after 30 days (see Table 4). At the first follow-up, 47.6% of surgical outcomes met standard criteria, including no wound dehiscence, no anterior chamber (AC) reaction, and a clear cornea. This improved to 59.5% at the third follow-up. Corneal haze was present in 11.9% of patients at the third follow-up. AC reaction and corneal haze at the first follow-up were more likely to correlate with poor visual outcomes than at the third follow-up.

**Table 4:** Summary of complications at follow-ups 1, 2 and 3 (n = 42) AC: anterior chamber

Complications	Number of patients (percentage)		
	Follow-up 1	Follow-up 2	Follow-up 3
Clear cornea, no wound, no AC reaction	20 (47.6)	32 (76.2)	25 (59.5)
Wound issues	02 (4.8)	0	0
Non-clear cornea	04 (9.5)	06 (14.3)	05 (11.9)
AC reaction	13 (31)	02 (4.8)	01 (2.4)
Lost to follow-up	03 (7.1)	02 (4.8)	11 (26.2)

The trends of complications showed that the cornea was clear in 76.2% of patients at the second follow-up, though there was a dip at the third follow-up, which could be attributed to a higher proportion of patients with clear corneas being lost to follow-up. The trend of the AC reaction decreased from 31% to 2.4% over time.

## Discussion

At baseline, the majority of our patients presented with marked visual impairment, and nearly 80% had visual acuity less than 6/60. This presentation was further aggravated by delayed surgical interventions due to long waiting times. In our case, over 70% waited more than 20 months for donor tissue. Postoperatively, over the six-month follow-up, there was a progressive improvement in vision, with the proportion achieving >6/60 increasing from merely 4.8% at the first follow-up to 68.7% by the third follow-up. While these outcomes validate a significant increase in visual acuity, they remain modest compared to the superior outcomes reported in settings with robust eye banking systems. Pramanik et al. (2006) reported that approximately 74% of keratoconus patients undergoing PK achieved a BCVA of ≥6/12 (20/40) by their last follow-up [12]. Our results align more closely with those observed in similar resource-constrained environments, as a similar study conducted in Bangladesh showed that 52.75% of the population had postoperative BCVA of >6/60 [13].

Our complication profile was encouraging, with relatively low postoperative ailments. We observed graft failure in 7.1% of patients by the end of the follow-up period, with no documented cases of graft rejection or infectious endophthalmitis. This is notably lower than the 17% graft rejection and failure rates reported by Yildirim et al. (2011) [14] and the 10.5% graft infection incidence found by Sung et al. (2015) [15]. Initial examinations included AC reactions in 31% of the patients at the first follow-up; however, these were resolved almost entirely by the third visit, as they persisted in only 2.4% of patients. Corneal haze was another unfavorable outcome observed in 11.9% of eyes at the final follow-up. Despite this, our complication rates are still comparable to those of many international studies. A Singapore cohort treated at a tertiary center reported postoperative rates of glaucoma (20.7%), graft rejection (18.2%), and late graft failure (9.4%) [16]. However, a 26.2% loss to follow-up by month six may have led to underrecognition of late-onset complications in our study.

Our study included a population of 64.3% male patients compared to 35.7% female patients, with a mean age of 33.90. Another retrospective, comparative study from Pakistan showed a similar mean age of 34.50 [6], which could be due to the fact that young individuals suffer more from corneal blindness as a consequence of corneal infections and trauma in LMICs.

Keratoconus with very low BCVA was our most common indication for PK. This finding is consistent with a study conducted in Turkey, showing keratoconus as the most prevalent indication for PK at 24.3% [17]. Corneal scarring was the second most common indication (31%) for PK. A similar study conducted in Bangladesh showed that out of 213 PK surgeries performed, 41.42% of them were done for corneal scarring [13]. A large study from Iran found that 29.56% of PK were performed for corneal scarring [18].

The biggest challenge we found was the lack of a domestic eye bank, which affected about 71% of our patients. Because we had to rely on imported corneas, this led to long waiting times; more than 70% had waited over 20 months for their surgery. By the time many patients finally underwent treatment, their disease had often progressed further, which might have worsened their preoperative condition.

This issue is not unique to our setting. In a large survey covering 112 countries, Gain et al. (2016) found that over half of the world’s population did not have timely access to corneal transplants, mostly due to the absence of local eye banks [19]. Waiting periods of one to two years were common. Similarly, Pineda (2015) noted that in places like India and Africa, limited eye banking infrastructure, shortages of trained staff, and low donor tissue availability all played a major role in why corneal blindness remained so prevalent [20].

Beyond logistics, we also faced technical limitations. In our study, critical tools like trephines were scarce in nearly 79% of cases. This aligns with what Oliva et al. (2012) reported, stating that the lack of basic surgical equipment was a major reason so few transplants were performed in similar regions [21].

Finally, we recorded a loss to follow-up of about 24% by six months, which was fairly substantial. This issue has also been highlighted in other studies from resource-limited areas, where distance, costs, or lack of patient education often made it hard to keep patients under long-term care.

The overall waiting time for undergoing a cornea transplant was 20 months in 73.8% of the patients in our study. This finding is significantly higher than the average waiting time reported in a study conducted in Brazil (127.19 days) [22] and in South Africa (280 days) [23]. This disparity may be attributed to the lack of locally available and accessible corneal transplant services and operational eye banks in underdeveloped countries. A lack of knowledge may also contribute to the smaller number of donors, as supported by a study conducted in Lahore, which concluded that a large proportion of the population does not have proper knowledge regarding corneal transplantation and eye donation [24]. Consequently, the number of available corneas is much lower than the number of patients needing transplants, leading to longer waiting periods.

The small sample size may be considered a primary limitation of this study, which may have confounded the statistical power. This hinders the generalization of the findings to the general population. Additionally, no patient randomization was performed, which raises the potential for selection bias. The patients were followed for up to six months, but this duration may not be sufficient for accurate determination of graft survival, particularly for non-domestic donors. Furthermore, one-fourth of the patients (n = 10, 23.8%) were lost to follow-up, which could potentially underestimate the rate of graft failure. We suggest a longer follow-up period to enable deeper insights into long-term complications and outcomes. Finally, the higher rate of loss to follow-up reduces confidence in the treatment outcomes of this study.

## Conclusions

This study emphasized challenges faced by LMICs in executing PK. The primary challenge was the loss to follow-up and delay in the availability of donor tissue, highlighting areas where the administrative and infrastructure shortcomings can be addressed. Another major challenge was the unavailability of surgical equipment. The positive impact of early corrective surgery is evident from the first and second follow-up results. The need for improvement in administrative measures, local infrastructure, and development of the local corneal bank will substantially enhance the PK services.
